# Axillary node metastasis from differentiated thyroid carcinoma with hürthle and signet ring cell differentiation. A case of disseminated thyroid cancer with peculiar histologic findings

**DOI:** 10.1186/1471-2407-12-55

**Published:** 2012-02-03

**Authors:** Maria Grazia Chiofalo, Nunzia Simona Losito, Franco Fulciniti, Sergio Venanzio Setola, Antonio Tommaselli, Ugo Marone, Maria Luisa Di Cecilia, Luciano Pezzullo

**Affiliations:** 1 Thyroid and Parathyroid Surgery Unit, Istituto Nazionale per lo Studio e la Cura dei Tumori, Fondazione G. Pascale, Via Mariano Semmola, 80131, Naples, Italy; 2 Department of Pathology, Istituto Nazionale per lo Studio e la Cura dei Tumori, Fondazione G. Pascale, Naples, Italy; 3 Radiology, Istituto Nazionale per lo Studio e la Cura dei Tumori, Fondazione G. Pascale, Naples, Italy; 4 Nuclear Medicine, Istituto Nazionale per lo Studio e la Cura dei Tumori, Fondazione G. Pascale, Naples, Italy; 5 Surgical Oncology, Istituto Nazionale per lo Studio e la Cura dei Tumori, Fondazione G. Pascale, Naples, Italy

**Keywords:** Thyroid cancer, Axillary lymph node metastasis, Distant metastases, Signet ring cells

## Abstract

**Background:**

Differentiated thyroid cancer is usually associated with an excellent prognosis and indolent course. Distant metastases are rare events at the onset of thyroid cancer. Among these presentations, metastasis to the axillary lymph nodes is even more unusual: only few cases were previously reported in the literature; there has been no report of axillary lymph node metastasis from follicular thyroid carcinoma. Axillary lymph node metastasis generally arises in the context of disseminated disease and carries an ominous prognosis.

**Case presentation:**

Here we present a case of axillary lymph node metastasis in the context of disseminated differentiated thyroid cancer. The patient underwent near total thyroidectomy and neck and axillary lymph node dissection. A histopathological diagnosis of poorly differentiated follicular carcinoma with "signet ring cells" and Hürthle cell features was established. The patient received radioactive iodine therapy and TSH suppression therapy. Subsequently his serum thyroglobulin level decreased to 44.000 ng/ml from over 100.000 ng/ml.

**Discussion and Conclusion:**

Currently there are only few reported cases of axillary node metastases from thyroid cancer, and to our knowledge, this is the first report on axillary lymph node metastasis from follicular thyroid carcinoma. "Signet ring cell" is a morphologic feature shared by both benign and, more rarely, malignant follicular thyroid neoplasm, and it generally correlates with an arrest in folliculogenesis. Our case is one of the rare "signet ring cells" carcinomas so far described.

## Background

Differentiated thyroid cancer is usually associated with an excellent prognosis and indolent course. Distant metastases are rare events at the onset of thyroid cancer. Their incidence is about 4%, with an overall risk which is lowest for papillary thyroid cancers (2%) and highest for follicular (11%) and Hürthle cell carcinomas (12%) [1]. Among these presentations, metastasis to the axillary lymph nodes is even more unusual. It generally arises in the context of disseminated disease and carries an ominous prognosis [2]. Herein, we present the first ever reported case of axillary lymph node metastasis from follicular thyroid carcinoma. Interestingly this presentation was associated with peculiar histologic features described, so far, only in four other cases of follicular-derived thyroid carcinoma.

## Case presentation

**Figure 1:**
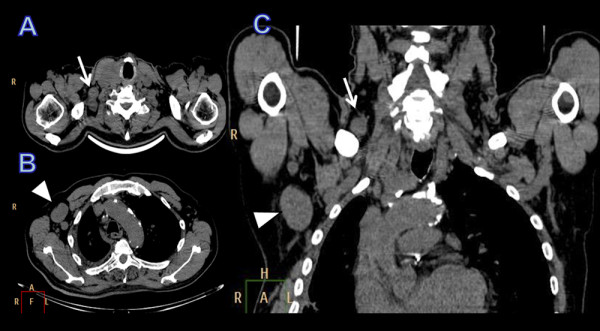
**CT imaging in axial (a, b) and coronal (c) plane: Lymph node metastasis in the right axilla (arrowhead) and in the supraclavicular fossa (arrow)**.

A 65-year-old man was referred on April 2010 to our surgical department with histologically documented metastatic thyroid carcinoma on biopsy samples taken from a pathological fracture of his left femur. He had already undergone orthopedic stabilization of the fracture followed by adjuvant radiotherapy. Staging work-up with Computed Tomography (CT) documented a tumor in the right lobe of the thyroid, multiple cervical supraclavicular and axillary lymph-nodes, multiple bone metastases and liver metastasis (Figure 1). Aspiration biopsy cytology of the neck and axillary lymph nodes showed malignant thyroid follicular epithelium-derived cells. Levels of serum thyroglobulin and anti-thyroglobulin antibodies were 102815,0 ng/mL and 727,4 IU/ml, respectively.

We treated the primary thyroid tumour with a near-total thyroidectomy including central plus right neck dissection and the addition of right axillary lymph node dissection.

On gross pathological examination, the thyroidectomy specimen weighted 74 g; the right lobe was enlarged, measuring 7.5 × 3.5 × 3 cm. The cutting surface showed a solid nodule, 3.8 × 2.5 cm in size at the apex of the right lobe. Microscopic examination of the thyroid tumor showed a follicular neoplasm with two growth patterns: micro- and macrofollicular areas alternating with predominantly cribriform and solid/insular areas. A thick, fibrous capsule was still evident around the nodule although it was completely invaded by the tumor; extensive vascular invasion, within and beyond the tumor capsule, was also observed.

**Figure 2:**
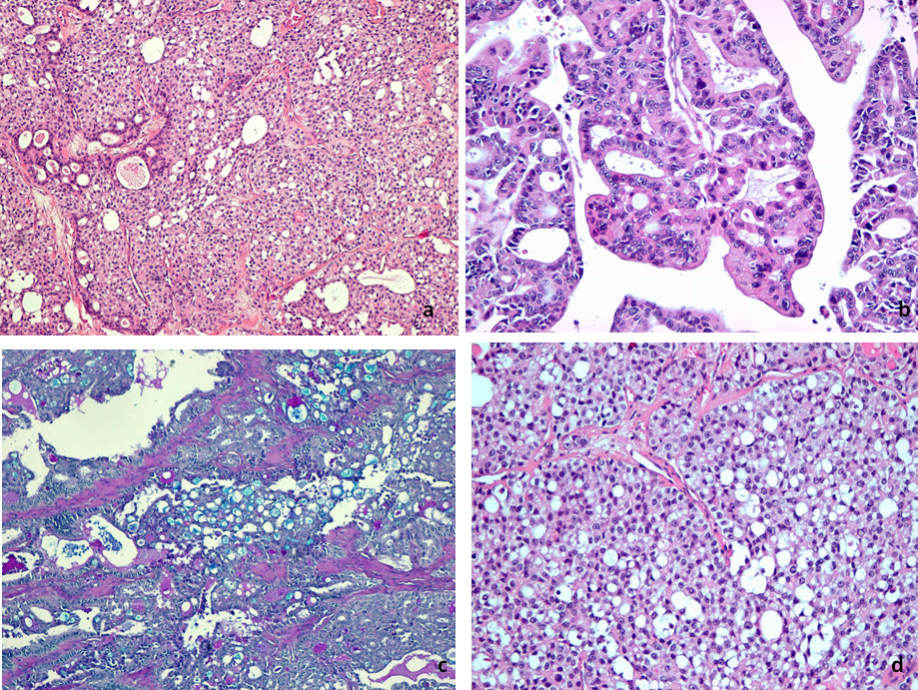
a) H/E 20x: Solid, poorly differentiated areas with focal follicular differentiation and many signet ring-like cells; b) H/E 40x: In axillary metastasis, follicular growth pattern was associated to pseudopapillary structures; more abundant oxyphilic cytoplasm and strong nuclear pleomorphism were evident; c) Alcian blu 20x: Signet ring cells contained Alcian blu positive mucous secretion; d) H/E 40x; Signet ring-like cells.

Histologically, the cuboid neoplastic cells had eosinophilic cytoplasm; a striking feature was represented by their frequent cytoplasmic vacuolization, with formation of "signet ring cells" having prominent cytoplasmic vacuoles with eccentric nuclei. Colloid-like material was found in micro-macrofollicular areas; focal mucoid secretion within glandular lumens and inside the "signet ring" cells too was highlighted by mucicarmine and Alcian-blue staining; 7/22 cervical lymph nodes, 5/23 axillary lymph nodes showed massive infiltration/total substitution by a follicular cell derived carcinoma with three peculiar cell types: 1) cylindrical, eosinophilic cells with apical snouts, arranged in follicle-like structures; 2) oxyphilic cells with abundant granular cytoplasm and strong nuclear pleomorphism; 3) cuboid cells with vesicular, round nuclei with deeply eosinophilic nucleoli, nuclear pseudoinclusions and a focal papillary growth pattern were also evident. Immunohistochemistry was performed using a peroxidase-antiperoxidase complex method. Neoplastic cells, both in the primary thyroid tumour and in the lymph node metastases, showed intense and diffuse staining for thyreoglobulin (TG) and thyroid transcription factor (TTF-1), while carcino-embryonic antigen (CEA) and CD15 stainings were negative. In areas with solid growth, p53, bcl2 and E-cadherin were intensively expressed, and a Ki 67 staining ratio of > 10% was found. A diagnosis of poorly differentiated follicular carcinoma with "signet ring cells" and Hürthle- cell features was made (Figure 2).

**Figure 3:**
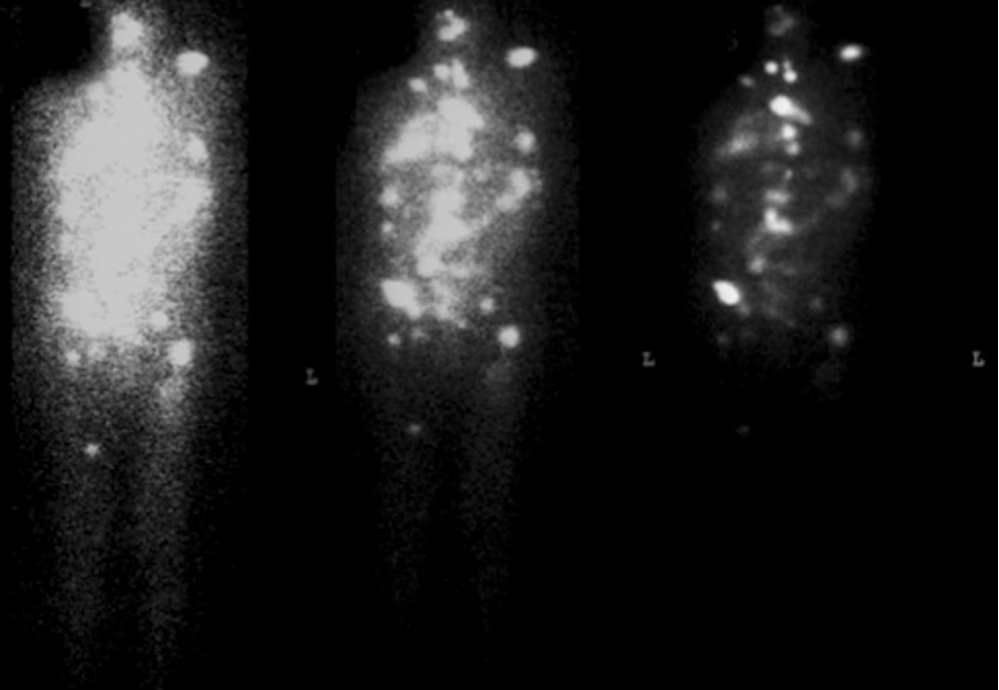
**Post-therapy radioiodine whole-body scan: Multiple areas of abnormal ^131^I uptake**.

The disease stage according to the AJCC criteria was IVc. The patient received 260 mCi of ^131^I in May 2010 and 260 mCi of ^131 ^I in October 2010. Radioiodine whole body scan performed after completion of treatment still detected multiple metastatic lesions (Figure 3). One year later, the patient is alive with persistent, stable disease and, on a background treatment with levotiroxine 150 mcg per day; TSH approaches 0.5 uIU/mL and thyroglobulin has lowered to 44.000 ng/ml.

## Discussion and Conclusion

Currently there are only few reported cases of metastases to the axillary lymph nodes in patients with thyroid cancer and, to our knowledge, this is the first report on metastasis to the axillary lymph nodes from follicular thyroid carcinoma. In the previously published cases, the primary tumours were mostly papillary thyroid carcinomas [2-9], and less frequently medullary thyroid carcinoma [7], sclerosing mucoepidermoid carcinoma with eosinophilia [10], mucin-producing poorly differentiated adenocarcinoma [11] and muco-epidermoid carcinoma [12]. In most of the previously reported cases, axillary lymph node metastases occurred in the context of disseminated disease, almost always as late recurrence. In our case it occurred at the presentation in the context of a disseminated follicular thyroid carcinoma. Some authors hypothesized that retrograde spread of the tumor into the axilla could be related to the blockage of the cervical lymphatic vessels by metastatic lymph nodes or by surgical manipulation [5]. This hypothesis seems consistent with our case. In our patient, in fact, there was a massive lymph nodes involvement of the neck levels IV and V. The axillary fat is contiguous with that of the inferolateral neck, so we hypothesize that the spread to the axillary lymph nodes could be related to the obstruction of lymphatic vessels. Follicular thyroid cancer has a high propensity to invade blood vessels and show-up as distant metastases, but the role of angiogenesis and lymphangiogenesis in thyroid cancer pathogenesis has not been clarified [13]. So, in the present case, it can't be established whether the axillary lymph node involvement is related to an hematogenous dissemination.

The pathological features of the neoplasm represent the second interesting aspect of our case. Primary mucinous carcinoma of the thyroid is extremely rare [14]. In the WHO classification, mucinous carcinoma of the thyroid is defined as a tumor characterized by clusters of neoplastic cells surrounded by extensive extracellular mucin deposit. On the other hand, many thyroid neoplasms, such as follicular adenoma, may produce mostly intracellular mucins, in particular the "signet ring cell" variant; moreover mucus may be produced also by carcinomas of papillary, follicular, medullary and mucoepidermoid subtypes. Mlynek identified mucin in 50% and 35% of papillary and follicular tumors, respectively, and emphasized that mucin production by follicular-derived tumors can be commoner than usually thought [15]. In the current case, "signet ring cells" and mucin-producing areas were frequently found.

To date, only four cases have been reported of follicular-derived thyroid carcinomas which show signet ring features: three by Schröder et al. [16] and one by Fellegara et al. in a poorly differentiated Hürthle cell carcinoma [17]. In a previous report, Gherardi et al. has presented convincing evidence that "signet ring cells" contained altered or abnormal thyroglobulin [18]. Fellegara and Rosai [17] have reported how vacuoles result from intracytoplasmic accumulation of thyroglobulin as demonstrated by immunohistochemistry, while they appear bordered by microvilli on electronic microscopy. This may suggest that they are intracytoplasmic follicular lumina rather than secretion vacuoles and represent the morphologic expression of an arrest in folliculogenesis [17,18].

In the present case, both thyroglobulin and mucoid secretion were demonstrated in the "signet-ring cells", according to previous reports [16]. Interestingly, microscopic examination of axillary metastases showed neoplastic cells with ample granular eosinophilic cytoplasm and pleomorphic nuclei, the typical features of Hürthle cell carcinomas, similarly to two previously reported cases of "signet ring cells" carcinoma [16,17]; on the other hand, the "signet ring cell" features, in our case, were prominent in the primary thyroid tumor, while they were lacking in metastases. "Signet-ring cells" are hence to be considered a morphologic feature shared by both benign and, more rarely, malignant follicular thyroid neoplasm, and probably reflect an arrest in folliculogenesis. Our case is one of the rare "signet ring cells" carcinomas so far described.

## Consent

Written informed consent was obtained from the patient for publication of this case report and any accompanying images.

## Competing interests

The authors declare that they have no competing interests.

## Authors' contributions

LP and MCG designed the study. MGC drafted and edited the manuscript. LP coordinated and helped to draft the manuscript. NSL and FF performed the histopathological, immunohistochemical examinations and participated in the draft the manuscript. LP, MGC, UM and MLD treated and observed the patient including follow-up. SVS acquired the radiographic pictures, and all authors read and approved of the final manuscript.

## Pre-publication history

The pre-publication history for this paper can be accessed here:

http://www.biomedcentral.com/1471-2407/12/55/prepub
